# Myocardial Perfusion SPECT Imaging in Dextrocardia with Situs Inversus: A Case Report

**DOI:** 10.7508/aojnmb.2016.02.008

**Published:** 2016

**Authors:** Olusegun Akinwale Ayeni, Nico Malan, Emmanuel Niiboye Hammond, Mboyo-Di-Tamba Heben Vangu

**Affiliations:** Division of Nuclear Medicine and Molecular Imaging, Department of Radiation Sciences, University of the Witwatersrand, Charlotte Maxeke Johannesburg Academic Hospital, Johannesburg, South Africa

**Keywords:** Dextrocardia, Image processing, Myocardial perfusion imaging, Situs inversus totalis

## Abstract

Dextrocardia is a cardiac positional anomaly in which the heart is located in the right hemithorax with its base-to-apex axis directed to the right and caudad. Situs inversus is an autosomal recessive disorder that causes organs in the chest and abdomen to be positioned in a mirror image from their normal position. Dextrocardia may occur in isolation or as part of situs inversus. Similarly, situs inversus may occur with or without dextrocardia. Situs inversus accompanied with dextrocardia (situs inversus totalis) is a rare congenital abnormality occurring in 0.01% of live births. Herein, we present the case of a 35-year-old man with previously diagnosed situs inversus totalis with mirror-image dextrocardia, referred to our facility for diagnosis of coronary artery disease (CAD). The incidence and presentation of CAD in patients with dextrocardia are similar to the normal population. However, considerable attention should be paid to the acquisition of myocardial perfusion scintigraphy and data processing/analysis in this group of patients. The present case highlights the distinctive applications and potential pitfalls of myocardial perfusion single-photon emission computed tomography (SPECT) imaging in patients with dextrocardia.

## Introduction

Dextrocardia is a cardiac positional anomaly in which the heart is located in the right hemithorax with its base-to-apex axis directed to the right and caudad (1). This disorder may occur independently or as part of situs inversus. Situs inversus is caused by an autosomal recessive disorder that causes organs in the chest and abdomen to be positioned in a mirror image from their normal position.

Situs inversus accompanied with dextrocardia is termed as “situs inversus totalis” and is recognized as a rare congenital abnormality, occurring in 0.01% of live births (2). The malposition is intrinsic to the heart and is not caused by extracardiac abnormalities. Optimal image acquisition of myocardial perfusion imaging (MPI) can be challenging in patients with dextrocardia.

Herein, we present the case of a 35-year-old man with dextrocardia (as part of situs inversus totalis), who was referred to our facility for the diagnosis of coronary artery disease (CAD), suspected on the account of a risk factor (hypertension) and history of recurrent chest pain.

## Case report

A 35-year-old man presented with a one-year history of typical chest pain and uncontrolled hypertension. The cardiac enzymes and electrocardiogram (ECG) were interpreted as normal. The patient was referred to the Nuclear Medicine Department for MPI studies in order to diagnose CAD.

**Figure 1:**
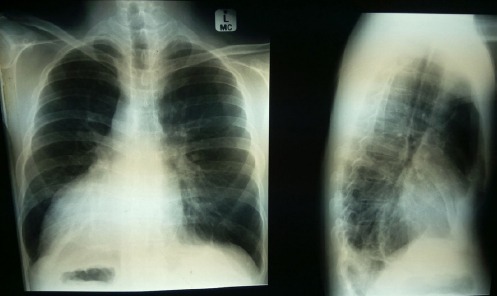
Chest X-ray showing a right-sided cardiac shadow along with cardiomegaly. Note the right-sided gastric bubble

The patient had suffered from hypertension since birth. He also had a history of situs inversus associated with dextrocardia, as well as polycystic kidneys diagnosed early in childhood. On cardiovascular examination, the apex was present in the sixth intercostal space on the right side, lateral to the mid-clavicular line. The acquired chest X-ray showed a right-sided cardiac silhouette with cardiomegaly and right gastric bubble (chest X-ray, Figure 1), supporting the diagnosis of situs inversus totalis.

A two-day rest-stress technetium (^99m^Tc) sestamibi protocol was applied. The patient underwent vasodilator stress testing with adenosine due to failure to reach the maximum age-predicted heart rate on physical exercise. For this purpose, 140 μg/kg/min of adenosine infusion was administered over 6 min, and 20 mCi (740 MBq) of ^99m^Tc sestamibi was injected at 3 min for the adenosine stress test.

ECG was performed with leads placed both conventionally and in reverse positions to account for dextrocardia. Changes with different lead placements are presented in Figures 2a and 2b, respectively. Moreover, tomographic images were acquired following the stress test with a dual-head gamma camera (Optima Discovery, General Electric), using a 64×64 matrix and 30 projections.

**Figure 2a:**
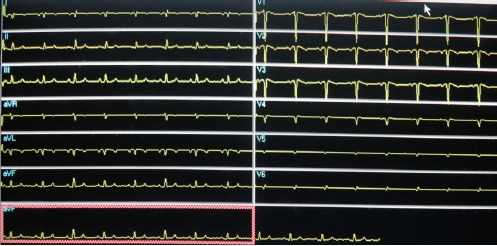
Electrocardiogram (ECG) with leads placed conventionally. Note that in lead I, the QRS complex is negative with inverted P-wave and T-wave. In precordial leads, there is reverse R-wave progression

**Figure 2b:**
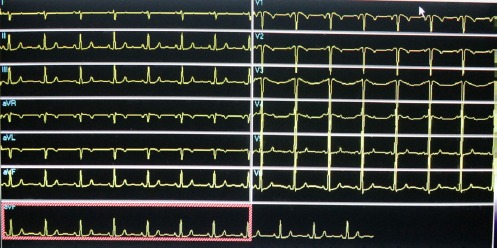
Electrocardiogram (ECG) with reversed leads. Note the normal R-wave progression in precordial leads

The images were acquired in the L mode over 180° counter-clockwise circular movements starting at 45° right posterior oblique projection and ending at 45° left anterior oblique projection instead of the standard right anterior oblique to left posterior oblique views in the head-out (feet-first supine) position.

Also, prone images were acquired to aid attenuation corrections. For the ECG-gated study, camera acquisition was triggered to R-wave and eight frames were collected per R-R interval. Rest images were obtained a day earlier with a similar acquisition protocol, following the tracer injection.

The acquired images were indicative of dextrocardia. An apparent lateral wall defect was detected because of the altered cardiac orientation (Figure 3a). Processing was repeated and particular attention was paid to the correct localization of the cardiac anatomy. An altered orientation was applied which revealed no evidence of infarction or adenosine-induced ischemia (Figure 3b). Therefore, identification of the exact position of the heart in cases with dextrocardia is crucial for the validity of examinations.

**Figure 3a:**
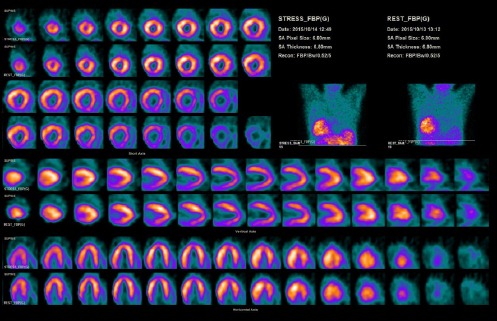
^99m^ Tc-MIBI SPECT images reconstructed with conventional processing, showing the right ventricle where the left ventricle is usually seen. Note the myocardial walls with a different localization

**Figure 3b:**
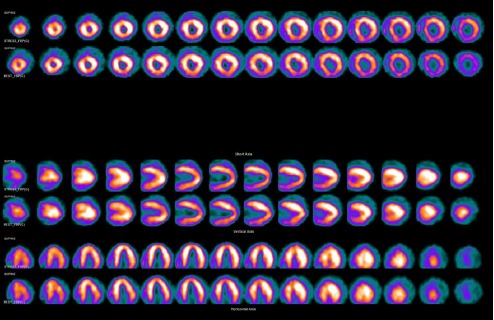
^99m^ Tc-MIBI SPECT images reconstructed with altered patient orientation during processing results in the conventional orientation of cardiac images

## Discussion

Dextrocardia with situs inversus is a rare congenital condition (prevalence of 1:10,000) in which the cardiac apex is positioned on the right side of the chest. Additionally, the position of heart chambers, as well as visceral organs such as the liver and spleen, is reversed. These individuals usually have a normal life expectancy and a similar chance to develop ischemic coronary disease as the general population (3-5).

MPI is a non-invasive imaging modality, commonly used to evaluate left ventricular wall perfusion, motion, and ejection fraction. There are a few studies in literature that have reported MPI studies in dextrocardia (2, 6-12). The term “mirror-image dextrocardia” is used in cases of dextrocardia with normal vascular anatomy, accompanied with situs inversus. In such cases, the anterior and inferior walls of the heart remain the same, whereas the septal and lateral walls change position in the right-left direction.

Knowledge of the exact position of the heart is important for the correct processing and validity of the examinations; therefore, it is essential to know how to acquire and analyze MPI in these patients. A 180° SPECT acquisition, ranging from -135° to +45°, is usually adopted during the imaging process, given the altered orientation of the heart within the thorax (13); in fact, orientation should also be considered during image processing.

Some gamma camera software programs have special acquisition and analysis protocols for dextrocardia, which must be followed to allow for correct interpretation of images. Significant errors may arise if patients with dextrocardia are not identified and normal MPI acquisition and analysis protocols are applied. In this regard, *Özdemir* and colleagues performed two different MPI acquisitions to understand the difference (8).

In the present case, although only one acquisition was obtained, the raw data were processed differently. The first reconstruction was performed using normal analysis parameters (Figure 3a), while in the second analysis, the projection image was acquired while the feet-first prone position was selected during analysis and the head-first supine position during imaging. This was to ensure that the heart and spatial position of the patient with dextrocardia were positioned like a “normal patient” (Figure 3b).

In this study, no significant difference was observed in terms of the gated data, which showed the imaging ejection fraction to be 29-30%. Moreover, global hypokinesis was observed in the processed data with conventional and altered patient orientations, as described above. The altered orientation in dextrocardia results in an apparent defect in the normal lateral wall.

A lateral wall defect may be reported even if the patient has normal left ventricular wall perfusion. This is because the wall is actually the septum, which normally shows relatively decreased perfusion, compared to other walls in normal MPI studies. True perfusion defects may also be encountered in incorrect walls. Therefore, it is important to review raw data and examine the whole data set of MP-SPECT images.

## Conclusion

Although rare, patients with dextrocardia may be referred for MP evaluations. Therefore, it is important to be prepared for accurate imaging, analysis, and report of MPI studies in these patients. If available, gamma camera software programs with special acquisition and analysis protocols for dextrocardia should be used. Specialists should always keep in mind the right-left mirroring of images during data processing, analysis, and report.
